# Automated medical image segmentation techniques

**DOI:** 10.4103/0971-6203.58777

**Published:** 2010

**Authors:** Neeraj Sharma, Lalit M. Aggarwal

**Affiliations:** School of Biomedical Engineering, Institute of Technology, Institute of Medical Sciences, Banaras Hindu University, Varanasi-221 005, UP, India; 1 Department of Radiotherapy and Radiation Medicine, Institute of Medical Sciences, Banaras Hindu University, Varanasi-221 005, UP, India

**Keywords:** Artificial intelligence techniques, computed tomography, magnetic resonance imaging, medical images artifacts, segmentation

## Abstract

Accurate segmentation of medical images is a key step in contouring during radiotherapy planning. Computed topography (CT) and Magnetic resonance (MR) imaging are the most widely used radiographic techniques in diagnosis, clinical studies and treatment planning. This review provides details of automated segmentation methods, specifically discussed in the context of CT and MR images. The motive is to discuss the problems encountered in segmentation of CT and MR images, and the relative merits and limitations of methods currently available for segmentation of medical images.

## Introduction

With increasing use of Computed topography (CT) and Magnetic resonance (MR) imaging for diagnosis, treatment planning and clinical studies, it has become almost compulsory to use computers to assist radiological experts in clinical diagnosis, treatment planning. Reliable algorithms are required for the delineation of anatomical structures and other regions of interest (ROI). The goals of computer-aided diagnosis (CAD) are:

- To automate the process so that large number of cases can be handled with the same accuracy i.e. the results are not affected as a result of fatigue, data overload or missing manual steps.
- To achieve fast and accurate results. Very high-speed computers are, now, available at modest costs, speeding up computer-based processing in the medical field.
- To support faster communication, wherein patient care can be extended to remote areas using information technology.

The techniques available for segmentation of medical images are specific to application, imaging modality and type of body part to be studied. For example, requirements of brain segmentation are different from those of thorax. The artifacts, which affect the brain image, are different - partial volume effect is more prominent in brain while in the thorax region it is motion artifact which is more prominent. Thus while selecting a segmentation algorithm one is required to consider all these aspects. The problems common to both CT and MR medical images are:

- Partial volume effect
- Different artifacts: example motion artifacts, ring artifacts, etc and
- Noise due to sensors and related electronic system.

There is no universal algorithm for segmentation of every medical image. Each imaging system has its own specific limitations. For example, in MR imaging (MRI) one has to take care of bias field noise (intensity in-homogeneities in the RF field). Of course, some methods are more general as compared to specialized algorithms and can be applied to a wider range of data. A brief survey of three generations of medical image segmentation techniques has been provided by D.J. Withey and Z.J. Koles.[1]

## Medical Imaging Modalities

Medical imaging is performed in various modalities, such as MRI, CT, ultrasound, positron emission tomography (PET), etc. In the present review, we are focusing primarily on the segmentation of MR and CT images only.

### 

#### Magnetic Resonance Imaging:

MR imaging is the most widely used technique in the field of radio imaging.[2,3] MR is a dynamic and flexible technology that allows achieving variable image contrast by using different pulse sequences and by changing the imaging parameters corresponding to longitudinal relaxation time (T1), and transverse relaxation time (T2), and signal intensities on T1 and T2 weighted images relate to specific tissue characteristics. The contrast on MR image is a factor dependent on pulse sequence parameters. The most common pulse sequences are T1- weighted and T2-weighted spin-echo sequences. MR imaging of the body is performed to get the structural details of brain, liver, chest, abdomen and pelvis which helps in diagnosis or monitoring the treatment.

### Brain MR Imaging:

MR is generally more sensitive in detecting brain abnormalities during the early stages of disease, and is excellent in early detection of cases of cerebral infarction, brain tumors, or infections. MR is particularly useful in detecting white matter disease, such as multiple sclerosis, progressive multifocal leukoencephalopathy, leukodystrophy, and post-infectious encephalitis. In contrast, CT scan fails to detect white matter abnormalities. In case of MR images of the brain, the primary determinants of signal intensity and contrast are the T1 and T2 relaxation times. The contrast is distinctly different on T1 and T2-weighted images. Also, brain pathologies have some common signal characteristics. Pathologic lesions can be separated into five major groups by their specific signal characteristics on the two basic images: T2- weighted, and T1-weighted [Table 1].

**Table 1:** Type of Pathology and its Contrast in T1 and T2 Weighted Image

*Pathology*	*Contrast in T2 weighted image*	*Contrast in T1 weighted image*
Solid Mass	Bright	Dark
Fat	Dark	Bright
Cyst	Bright	Dark
Acute and chronic blood	Dark	Gray
Sub acute blood	Bright	Bright

### MR Liver Imaging:

MR provides outstanding intrinsic soft contrast that can enhance subtle differences between normal and pathologic tissues and tissues of different histologic subtypes. Nonionizing radiation is used and MRI contrast agents are not nephro-toxic. MRI images may be acquired with multiplanar capabilities which are especially useful in depicting various anatomic relationships. MRI system is specifically used in characterization of metastases and primary liver tumors e.g., benign lesions such as focal nodular hyperplasia (FNH), adenoma, hemangioma and malignant lesions (cancer) such as hepatocellular carcinomas (HCC).

**Chest MRI** is used to detect following disorders: thymus tumor, lung masses, esophageal mass, other masses (aggregations of cells) or tumors of the chest, abnormal lymph nodes, swollen glands and enlarged lymph nodes in any location of the chest, staging of tumors including invasion of blood vessels, alveolar bullae (COPD), bronchial abnormalities, bronchiectasis, cystic lung lesions, pleural abnormalities, including thickening or pleural effusion, abnormal pulmonary vessels, aortic stenosis, etc.

**Abdominal MRI** may reveal many medical conditions, including: abscess, acute tubular necrosis, adrenal masses, cancer, enlarged spleen or liver, gallbladder or bile duct problems, gallstones, bile duct stones, hemangiomas, kidney infection, kidney damage, lymphadenopathy, obstructed venacava, pancreatic cancer, tumor of the gallbladder, abdominal aortic aneurysm, ovarian cancer, etc.

MR Angiography is used to detect blockages or enlargements of blood vessels, including the aorta, renal arteries, and arteries in the legs, renal arterial obstruction, renal vein thrombosis, etc.

The following artifacts are present in MR imaging:

1. Partial Volume
2. RF Noise
3. Intensity inhomogeneity
4. Gradient
5. Motion
6. Wrap Around
7. Gibbs Ringing
8. Susceptibility

**Figure 1:**
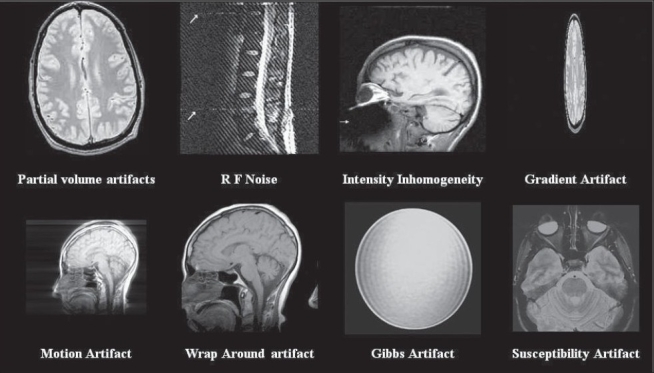
Artifacts in MR Imaging

These MR imaging artifacts are shown in figure 1 respectively.

#### The main advantages of MR imaging system are:.

- It has an excellent capability for soft tissue imaging
- It has very high resolution of the order of 1mm cubic voxels
- It has high signal to noise ratio
- Multi channel images with variable contrast can be achieved by using different pulse sequences; this can be further utilized for segmenting and classifying different structures.

#### Disadvantages of MR imaging:

- MR acquisition takes considerably longer time as compared to CT and
- In case of MR it is more difficult to obtain uniform image quality.

#### Computed Tomography Imaging:

The word tomography is derived from two Greek words; tomos, which means slice or section, and graphia, which means description. CT scan is an imaging modality which uses X-rays to obtain structural and functional information about the human body. The CT image is the reconstructed image and is reconstructed on the basis of X-ray absorption profile. X-rays are electromagnetic waves and used in diagnosis based on its property that all matters and tissues differ in their ability to absorb X-rays.[2] Dense tissues such as the bones appear white on a CT film while soft tissues such as the brain or liver appear gray. The cavities filled with air such as lungs appear black. CT performs better in cases of trauma and emergent situations. It provides better bone detail and has high sensitivity for acute hemorrhage. CT has become an important tool in medical imaging to supplement X-rays, medical ultrasonography (USG) and MR imaging. Although it is still quite expensive, it is the gold standard in the diagnosis of a large number of different disease entities. It is, more recently, being used in early screening of diseases, for example CT colonography for patients with a high risk of colon cancer.

CT scans are particularly used in imaging and the diagnosis of following body parts: brain, liver, chest, abdomen and pelvis, spine and also for CT based angiography.

### Brain CT Imaging:

In case of brain imaging, CT scans are typically used to detect: bleeding, brain damage and skull fracture in patients with head injuries; bleeding caused by a ruptured or leaking aneurysm in a patient with a sudden severe headache, blood clot or bleeding within the brain shortly after a patient exhibits symptoms of a stroke, brain tumors, cyst, diseases related to malformations of the skull, enlarged brain cavities (ventricles).

CT scanning is fast and simple, provides more detailed information on head injuries, and stroke; can reveal internal injuries and bleeding quick enough to help save lives in emergency cases.

### Liver CT Imaging:

In case of liver imaging, CT is the most commonly used imaging technique for evaluation of hepatic lesions. Large hepatocellular carcinomas tend to be heterogeneous, and may demonstrate a typical mosaic appearance on CT. There are relative advantages and disadvantages of both hepatic MR and CT. In general, CT is less costly than MR, more readily available, and most radiologists and many referring physicians have a relatively high degree of confidence in looking at CT images. Some studies, however, have found that CT is less sensitive and specific than MR for detection and characterization of focal hepatic disease.

### Chest Imaging:

Chest CT is used to detect: tumors in lungs, pneumonia, tuberculosis, emphysema, diffuse interstitial lung diseases, inflammation or other diseases of pleura, the membrane covering the lungs.

### Abdomen and Pelvis CT Imaging:

It is used to detect: abscesses in abdomen, inflamed colon, cancers of (i) colon, (ii) liver and (iii) pancreas, pancreatitis, lymphoma, diverticulitis, appendicitis.

### Spine CT Imaging:

It is used to detect various types of tumors in vertebral column, herniated inter-vertebral disk, fractures and other injuries and measure bone density and level of osteoporosis.

CT based angiography is used to identify a small aneurysm or arteriovenous malformation inside the brain, detect thrombosis in veins, atherosclerotic disease in the carotid artery and indicate disease in renal artery.

The artifacts present in CT imaging are:[4]

**Figure 2:**
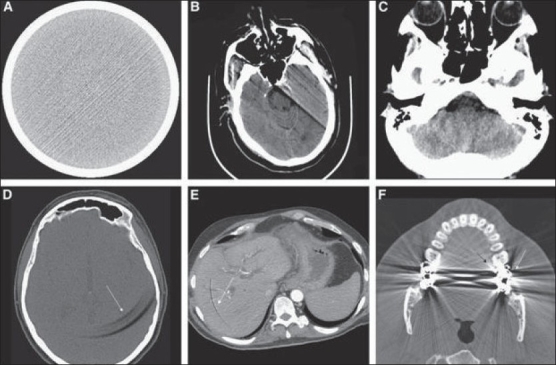
Examples of CT Artifacts: (A) Streak (B) Motion (C) Beam-hardening (D-E) Ring (F) Bloom [4]

1) partial volume effect [Figure 1a], 2) streak artifacts, 3) motion artifacts, 4) beam hardening artifacts, 5) ring artifacts, and 6) bloom artifacts.

These artifacts are shown in Figure 2.

#### Advantages of CT imaging include:

- Less expense and wide availablity
- High spatial resolution with modern multi-slice scanners
- Short scan time
- Higher sensitivity than MR for sub-arachnoids hemorrhage
- Higher sensitivity in detecting intra-cranial calcifications

#### Disadvantages of CT imaging system are:

- Inferior soft tissue contrast compared to MRI as it is X-ray-based
- Radiation exposure

Despite the disadvantages, CT scans are extensively used in the radiographic study of brain, liver and thorax.

### Representation of Medical Images:

Images are presented in 2-D as well as in 3-D domain. In the 2-D domain each element is called pixel, while in 3-D domain it is called voxel. In some cases we represent 3-D images as a sequential series of 2-D slices. The advantages associated with this type of representation include requirement of lower computational complexity and lesser memory.[5,6]

## Segmentation

Segmentation is the process dividing an image into regions with similar properties such as gray level, color, texture, brightness, and contrast.[7–9] The role of segmentation is to subdivide the objects in an image; in case of medical image segmentation the aim is to:

- Study anatomical structure
- Identify Region of Interest i.e. locate tumor, lesion and other abnormalities
- Measure tissue volume to measure growth of tumor (also decrease in size of tumor with treatment)
- Help in treatment planning prior to radiation therapy; in radiation dose calculation

Automatic segmentation of medical images is a difficult task as medical images are complex in nature and rarely have any simple linear feature. Further, the output of segmentation algorithm is affected due to

- partial volume effect.
- intensity inhomogeneity
- presence of artifacts
- closeness in gray level of different soft tissue

Artifacts present in MR and CT images can be divided into three categories on the basis of image processing technique needed to rectify them: (i) artifacts needing appropriate filtering technique. For example, noise artifact, susceptibility artifact and presence of nonsharp edges in the image (ii) artifact needing appropriate image restoration techniques for example motion artifacts and (iii) artifact needing specific algorithm are; partial volume, intensity inhomogeneity.

Although a number of algorithms have been proposed in the field of medical image segmentation, medical image segmentation continues to be a complex and challenging problem. Different researchers have done the classification of segmentation techniques in one or another way.[1,10] At present, from the medical image processing point of view we have done the classification of segmentation techniques on the basis of gray level based and textural feature based techniques. Further, we consider artificial intelligence as tools to optimize these basic techniques to achieve accurate segmentation results. Thus, the broad classification of techniques available for segmentation of an image classified into two classes is as follows:

### Methods based on gray Level features

- Amplitude segmentation based on histogram features[11]
- Edge based segmentation
- Region based segmentation[12]

### Methods based on texture features[13,14]

### Method based on gray level features

### Amplitude segmentation based on histogram features

This includes segmentation of an image based on thresholding of histogram features and gray level thresholding is perhaps the simplest example of this technique. This is particularly suitable for an image with region or object of uniform brightness placed against a back ground of different gray level, A threshold can be applied to segment the object and background. Mathematically the threshold can be defined as follows.

(1) $$ri,j=\{\begin{array}{cc} 1 & pi,j\geq T\\ 0 & pi,j≺T \end{array}$$

Where *r_i, j_* is the resulting pixel at co-ordinate *(i, j), p_i, j_* is the pixel of input image and *T* is the value of threshold.

Equation 1 gives good results for segmentation of image with bi-modal histogram and fails in the case of an image with multi-modal histogram. Thresholding operation, defined by equation 1 is very basic and simple, and works well only when the object and background have uniform brightness of distinct gray level values respectively. This simple threshold operation does not work well at segmentation of images with multiple objects each having distinct gray level value varying over a band of values. To overcome this limitation, band thresholding based multiple thresholding operation is applied as follows:

(2) $$\begin{array}{cc} r_{i,j} & =1{\text{ for T}}_{1}<p_{i,j}\leq {\text{T}}_{2}\\ & =2{\text{ for T}}_{2}<p_{i,j}\leq {\text{T}}_{3}\\ & =3{\text{ for T}}_{3}<p_{i,j}\leq {\text{T}}_{4}\\ & ={\text{k for T}}_{\text{k}}<p_{i,j}\leq {\text{T}}_{\text{k}}\\ & =0\text{ otherwise} \end{array}$$

Here, the K^th^ band is corresponding to object/region having pixel values in the range of T_k_ to T_k+1_ where T_k_ is the lower limit of gray level and T_k+1_ is the upper limit of Gray level band.

**Figure 3:**
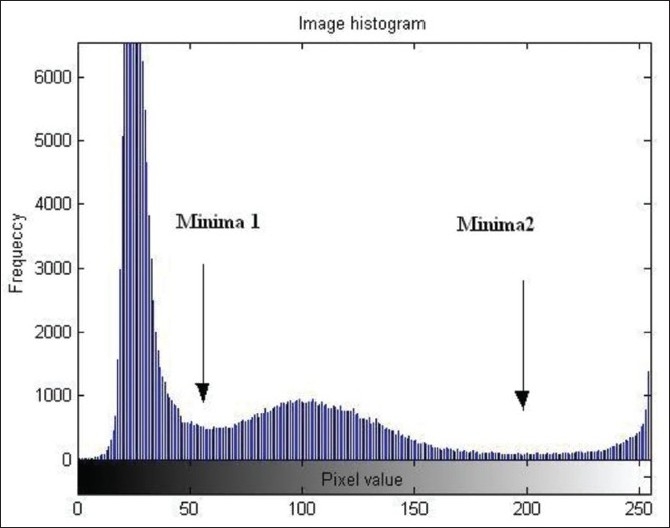
Image Histogram (three peaks separated by two minima)

For application of thresholding based segmentation technique, it is required to apply the correct threshold values in order to achieve proper segmentation results, otherwise results are poor. The histogram of an image is particularly used to determine the value of threshold. The histogram of abdomen CT image is shown in Figure 3. There are three peaks (maxima) separated by two minima. The values of these minima are selected as threshold for segmentation of image; the original Abdomen CT image and corresponding segmentation result are shown in figures 4a and 4b respectively.

**Figure 4a:**
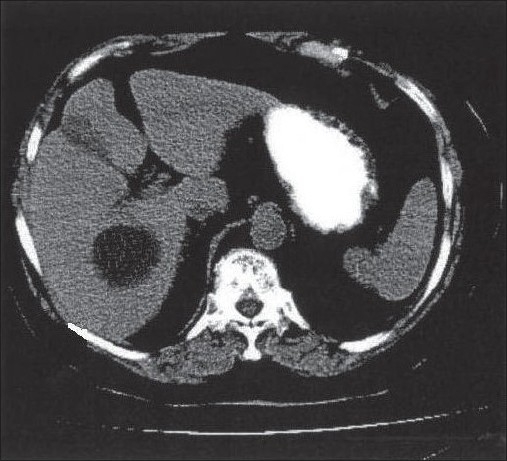
Original Abdomen CT Image

**Figure 4b:**
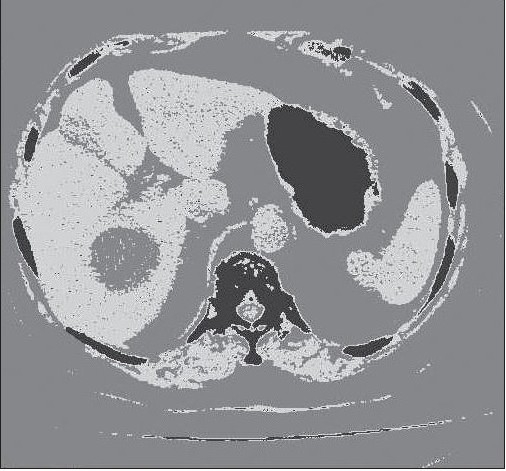
Segmentation of Abdomen (CT image using threshold technique)

Object 1 belongs (0 to 55)

Object 2 belongs (55 to 200)

Object 3 belongs (200 to 255)

For histogram-based optimal segmentation of images numbers of methods have been proposed by different researchers.[11,15–17] Frank *et al*,[17] have used optimal threshold selection method for segmentation of T1 weighted MR brain image to segment gray matter, white matter, and cerebro spinal fluid.

### Limitations

Selection of proper values of threshold is quite difficult. Performance is affected in presence of artifacts.

### Edge based segmentation

Edge based segmentation is the most common method based on detection of edges i.e. boundaries which separate distinct regions. Edge detection method is based on marking of discontinuities in gray level, color etc., and often these edges represent boundaries between objects. This method divides an image on the basis of boundaries.

Number of edge detecting operators based on gradient (derivative) function are available e.g. Prewitt, Sobel, Roberts (1^st^ derivative type) and Laplacian (2^nd^ derivative type), Canny, Marr-Hilclrath edge detector. Further, in edge based segmentation method, it is required to build the border by combining the detected edges into a edge chain in this process the spurious, or fake edges, weak edges are removed by thresholding operation. The different edge based segmentation algorithms are:

- Edge relaxation,[18]
- Border detection method,[19–21]
- Hough transform based[22,23]

The generalized algorithm for edge based segmentation has the following steps.

1. Apply the derivative operator to detect edges of the image
2. Measure the strength of edges by measuring amplitude of the gradient
3. Retain all edge having magnitude greater than threshold T (removal of weak edge)
4. Find the position of crack edges; the crack edge is either retained or rejected based on the confidence it receives from it predecessor and successor edges
5. Step 3 and 4 are repeated with different values of threshold so as to find out the closed boundaries; segmentation of an image is achieved

**Figure 5:**
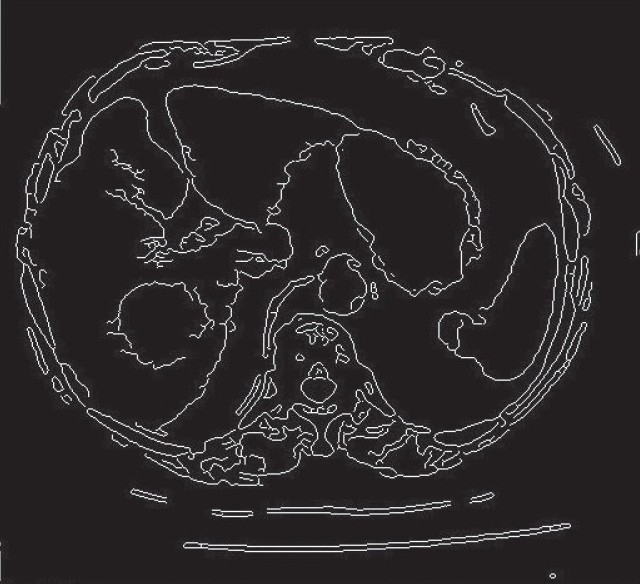
Result of Edge-based Segmentation of Abdomen (CT image)

Figure 5 shows the result of edge based segmentation of abdomen CT image [Figure 4a], in the present result canny edge detector has been employed.

The limitations of edge based method are:

- Performance is affected by the presence of noise
- fake edges and weak edges may be present in the detected edge image which may have a negative influence on segmentation results
- Edge detection techniques are required to be used in conjunction with region-based technique for complete segmentation.

### Region based segmentation

Region based methods are based on the principle of homogeneity - pixels with similar properties are clustered together to form a homogenous region. The criteria for homogeneity is most of the time gray level of pixels[24] and this criteria can be specified by following conditions

R1∪.R2∪.R3∪....∪.Ri=I

where *R_1_, R_2_, R_3_, …R_i_* are the region in the image *I*,

and further, $\begin{array}{l} R_{1}\cap R_{2}\cap R_{3}\cap ...\cap R_{i}=0 \end{array}$

This is as per the set theory of homogeneity.

Region based segmentation is further divided into three types based on the principle of region growing:

- Region merging
- Region splitting
- Split and merge

### Region merging

- In this method some seeding points are required to initialize the process, the segmentation results are dependent on the choice of seeds.
- Regions are grown iteratively by merging the neighboring pixels depending upon the merging criterion.
- This process is continued until all pixels are assigned to their respective regions as per merging criterion.

### Region splitting:

Its principle is just opposite to region merging and whole image is continuously split until no further splitting of a region is possible.

### Split and merge method:

This is the combination of splits and merges utilizing the advantage of the two methods. This method is based on quad quadrant tree representation of data whereby image segment is split into four quadrants provided the original segment is non-uniform in properties. After this the four neighboring squares are merged depending on the uniformity of the region (segments). This split and merge process is continued until no further split and merge is possible.

The algorithm for split and merge follows the following steps.

1. Define homogeneity criterion. Break image into four square quadrants
2. If any resultant square is not homogeneous split it further into four quadrants
3. At each level merge the two or more neighboring regions satisfying the condition of homogeneity
4. Continue the split and merge until no further split and merge of region is possible

Apart from the above-mentioned techniques watershed segmentation based on the concept of topography and hydrography is also a region-based segmentation.[25–28]

**Figure 6:**
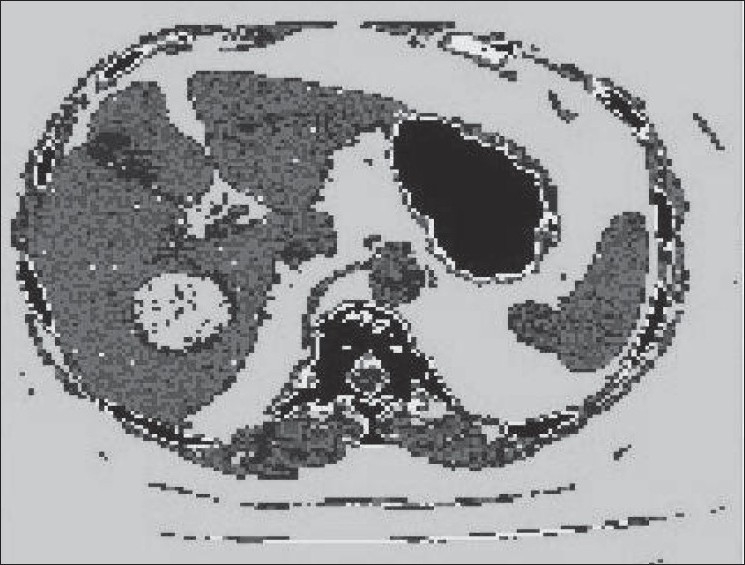
Segmentation of Abdomen (CT image using region based technique)

Figure 6 shows the result of region based segmentation of abdomen CT image [Figure 4a]. K-mean clustering algorithm has been used in present case to cluster the pixels having similar gray levels.

The limitation of region based segmentation is that there are chances of under segmentation and over segmentation of regions in the image. However, this problem can be rectified in two ways

- By optimally selecting the criterion for segmentation, for this several algorithm utilizing artificial intelligence techniques have been developed.
- By combining region based approach with edge based approach.[12,29]

### Method based on the textural features

Textural features of image are important from image segmentation and classification point of view. Different researchers have used these features to achieve image segmentation, classification, and both segmentation as well as classification. The aim of texture based segmentation method is to subdivide the image into region having different texture properties, while in classification the aim is to classify the regions which have already been segmented by one or other method.

#### Definition of texture:

Texture is defined as something consisting of mutually related elements.[24] A texture may be fine coarse, smooth, or grained depending upon its tone and structure. While tone is based on pixel intensity properties, structure is the spatial relationship between pixels.[30,31] Further texture can be defined as the spatial arrangements of texture primitives or texture elements (also called as textone), arranged in more or less periodic manner. Texture primitive is a group of pixels representing the simplest or basic sub pattern and follows three main approaches for texture feature extraction based on the type of approach used.

1. Statistical approach
2. Syntactic or structural approach and
3. Spectral approach

In case of statistical approach, texture is defined by a set of statistically extracted features represented as vector in multidimensional feature space. The statistical features could be based on first-order, second-order or higher-order statistics of gray level of an image. The feature vector so generated from patterns is assigned to their specific class by probabilistic or deterministic decision algorithm.[32] In case of syntactic approach, texture is defined by texture primitives which are spatially organized according to placement rules to generate complete pattern.

In syntactic feature based pattern recognition, a formal analogy is drawn between the structural pattern and the syntax of language.[33]

In spectral method, textures are defined by spatial frequencies and evaluated by autocorrelation function of a texture.

Some methods available for textural feature extraction and classification based on the above approaches are: co-occurrence matrix method based on statistical description of gray level of an image,[34,35] gray level run length method,[36] fractal texture description method,[37] syntactic method[38] and Fourier filter method.[39]

Comparing the above-mentioned three approaches; spectral frequency-based methods are less efficient while statistical methods are particularly useful for random patterns/textures and for complex patterns, syntactic or structural methods give better results.

Texture based methods as best suited for segmentation of medical image, when compared to segmentation of medical image using simple gray level based methods.[40–42]

### Other approaches of segmentation

Apart from the above methods, the following two methods of image segmentation are also available.

- Model based segmentation and
- Atlas based segmentation.

### Model based segmentation:

The basic approach is that the structure of organs has a repetitive form of geometry and can be modeled probabilistically for variation of shape and geometry. This can be used as constraint while segmenting the image and involves:

1. registration of the training data.
2. probabilistic representation of variation of registered data.
3. statistical influence between model and image.

Model based methods of segmentation involve active shape and appearance model, deformable models and level-set based models.

### Disadvantages

1. They require manual interaction to place an initial model and choose appropriate parameters.
2. Standard deformable models can also exhibit poor convergence to concave boundaries.[10]

**Atlas based segmentation approaches** are the most frequently used and powerful approaches in the field of medical image segmentation. In this, information on anatomy, shape, size, and features of different, organs, soft tissues is compiled in the form of atlas or look up table (LUT). Atlas guided approaches are similar to co-relation approaches and the plus point of atlas based approaches is - it performs segmentation and classification in one go. Atlas based segmentation approaches are among the third-generation algorithms. There are indications that certain atlas based methods can compete with manual segmentations although atlas selection, atlas registration procedure, and the manual tracing protocol used in atlas formation are factors that can affect performance.[1] However, they face limitations in segmenting complex structure with variable shape, size, and properties and expert knowledge is required in building the database.

### Artificial Intelligence Tools for Segmentation and Classification

**Automatic segmentation** methods have been based on artificial intelligence (AI) based techniques. AI techniques can be classified as supervised and unsupervised. Supervised segmentation requires operator interaction throughout the segmentation process whereas unsupervised methods generally require operator involvement only after segmentation is complete. Unsupervised methods are preferred to ensure a reproducible result[43]; however, operator interaction is still required for error correction in the event of an inadequate result.[44]

### Supervised methods

In the supervised category, we can place mostly Artificial Neural Network (ANN) based algorithms. ANN is composed of large number of interconnected processing elements (artificial neurons) working in unison to solve specific problems. The main advantages of ANN are:

- ability to learn adaptively, using training data to solve complex problems.
- capability of self-organization; it can create its own organization depending upon the information it receives during learning time
- capability of performance in real time because of parallel configuration

In case of ANN, learning is achieved by the adaptation of weights and bias of the neurons with respect to the training procedure and training data. ANN has been widely used for segmentation and classification purposes in both supervised and unsupervised modes.[45–47] Although a variety of different neural network based algorithms have been developed for texture based segmentation and classification with good classification accuracy,[47,48] most of these texture classifier algorithms require extensive supervision, training; their performance is sensitive to training parameters and is adversely affected in the presence of noise. At times supervised image segmentation and classification methods become very expensive, difficult and even impossible to correctly select and label the training data with its true category.[49] Training is the main requirement of many ANN based algorithms where the classifiers need to be trained before it can be applied to segmentation and classification problem. Further, for different data sets, analysis of different images of different type and format, the whole effort of selecting training data set and training is required to be redone.

### Unsupervised methods

Most of the unsupervised algorithms are cluster based and not dependent on training and training data. The two commonly used algorithms for clustering are K-mean or Hard C-mean and Fuzzy C-means.[50] K-means algorithm produces results that correspond to hard segmentation while fuzzy C-mean produces soft segmentation which can be converted into hard segmentation by allowing the pixels to have membership of cluster in which they have maximum value of membership coefficients.

In clustering, the aim is to construct decision boundaries based on unlabeled training data.[49] Clustering is the process of finding natural grouping clusters in multidimensional feature space. It is difficult because clusters of different shapes and sizes can occur in multidimensional feature space. A number of functional definitions of clusters have been proposed: Patterns within a cluster are more similar to each other than patterns belonging to different clusters.[49] Image segmentation may be considered a clustering[51–53] process in which the pixels are classified into the attribute regions based on the texture feature vector calculated around the pixel local neighborhood. Fuzzy clustering is a good method of classifying collection of data point to reside in multiple clusters with different degrees of membership (fuzzy c mean algorithm).[50]

However, the main limitations of fuzzy clustering algorithm are: (a) sensitivity to initial partition matrix (b) stopping criterion (c) solution may get stuck at local minima. Hence, clustering techniques may not result in optimal solution and there is no best clustering algorithm for a particular application. A number of different algorithms are required to be tried to find the best one.

### Segmentation of CT and MR Images

Segmentation of CT and MR images involves three main image related problems; *noise* that can alter the intensity of a pixel such that its classification becomes uncertain, *intensity inhomogeneity* where the intensity level of a single tissue class varies gradually over the extent of the image, and images have finite pixel size and are subject to *partial volume averaging* where individual pixel volumes contain a mixture of tissue classes so that the intensity of a pixel in the image may not be consistent with any one class.

Some methods available for CT image segmentation are:

- threshold based[54]
- region based[55]
- deformable models based[56–57]
- fuzzy based[58,59]
- neural network based[45–47,60]

### Methods available for MR image segmentation:

MR imaging is specifically used in brain imaging and thus lot of research work has been done particularly in the areas of MR brain image segmentation.[61–64] The main goal in brain MR segmentation is to segment gray matter, white matter and cerebrospinal fluid. Segmentation is also used to find out the regions corresponding to lesions tumors, cyst, edema, and other pathologies and for this mostly T1- weighted images are used.

Most of the segmentation methods available for CT and MR images segmentation are intensity based i.e. gray level based hence; the segmentation results are affected by (1) intensity in-homogeneities and (2) partial volume effects. Accordingly, different researchers have proposed methods for correction of these problems.

### Intensity In-homogeneity Correction:

In MRI intensity, inhomogeneity artifacts cause shading effect to appear over the images.[65] As a result, they affect the segmentation result while using simple gray level based segmentation techniques. One way to rectify these intensity inhomogeneity artifacts is by using image enhancement techniques.[66,67] Further, for this segmentation, techniques based on statistical methods[68,69] and fuzzy methods,[58,70–73] which gives soft segmentation results are particularly useful in overcoming the above mentioned limitation. A review of methods for correction of intensity inhomogeneity artifact in MR images has been presented by Vovk *et al*.[74]

### Partial Volume Effect Correction:

When multiple tissues contribute to single pixel or voxel the resultant image is blurred at boundaries of the different region or object and this effect is called as partial volume effect. To deal with partial volume effect, soft segmentation is a good option. In soft segmentation we allow the region or classes to overlap i.e. pixels are allowed to have multiple memberships with varying degree of membership coefficient in different regions. In hard segmentation, we do not allow overlapping of the segmented region and the pixels are forced to reside in the region in which they are having maximum membership. Thus soft segmentation retains more information about the original image by allowing the pixel to have membership in the multiple regions.

For hard segmentation the pixel is having binary membership defined as

mk,j={1ifj∈Rk0otherwise

Where *j* is j^th^ pixel belongs to image (I), and *m_k,j_* is the membership function of j^th^ pixel in region R_k_. Similarly for soft segmentation the pixel has multiple memberships in different regions and the membership function must satisfy the following constraints:

0≤mk,j≤1 for all k, jand ∑k=1Nmk,j=1  ∀j

where N = total number of segmented regions in the image (I)

From the value of membership of pixel j in R_k_ (k^th^ region) one can measure how strongly the pixel belongs to the region; more the membership value more strongly it is the member of region R_k_. At the border region the pixel can have different membership in different regions.

**Figure 7:**
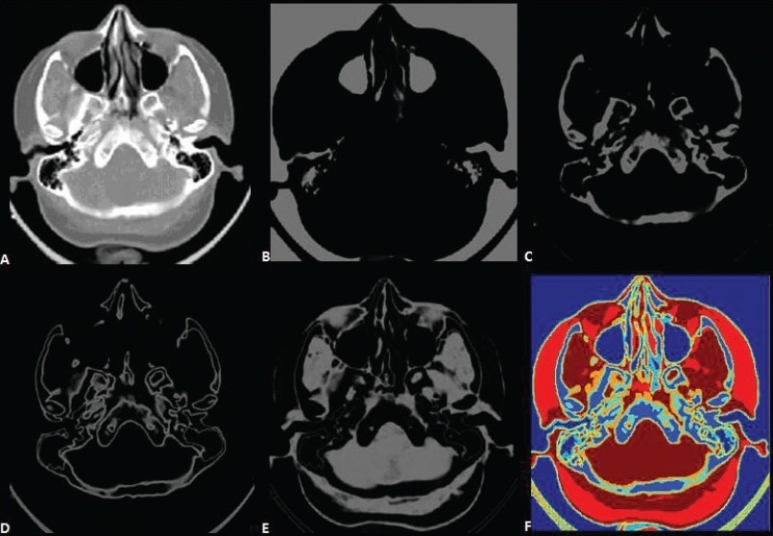
Individual Segments of Brain CT Image (A) Original (B-E) Individual segments (F) Segmented image in Pseudo Color

Fuzzy clustering is an excellent method for soft segmentation[58,72] and the most widely used unsupervised algorithm for segmentation of both CT and MR images. Soft segmentation based membership can be converted to hard segmentation by allowing the pixel to have the value of membership function one (1) corresponding to the region for which it has highest membership value. Figure 7A shows brain CT image; individual segments of this image obtained using simulated annealing based fuzzy-c-means algorithm[58] are shown in Figure 7B–7E and Figure 7F shows segmented image in pseudo color.

## Conclusion

Computer-aided segmentation is a key step finding application in computer aided diagnosis, clinical studies, and treatment planning. In recent years a wide variety of approaches have been proposed to segment CT and MR images having their own merits and limitations. The present review provides the basics of segmentation approaches and their respective features.

The approaches for image segmentation discussed in this review can be ranked on the basis of applicability, suitability, performance, and computational cost. Segmentation techniques based on gray level techniques such as thresholding, and region based techniques are the simplest techniques and find limited applications. However, their performance can be improved by integrating them with artificial intelligence techniques. Techniques based on textural features utilizing atlas or look-up-table have excellent results on medical image segmentation. However, they need expert knowledge in building the atlas. The limitation of atlas based technique is that under certain circumstances it becomes difficult to correctly select and label data; has difficulties in segmenting complex structure with variable shape, size, and properties. In such situations it is better to use unsupervised methods such as fuzzy-c-means algorithm.

A variety of different neural network-based algorithms are also available for texture-based segmentation and classification having good accuracy. However, most of these neural network-based algorithms require extensive supervision and training and their performance depends upon the training method and data used in training. Finally, it is desired from medical image segmentation and classification algorithms that they must have the following features: a) accuracy, b) reliability, c) repeatability, d) robustness and e) least dependency on the operator.
